# Environmental Thermal Stress Induces Neuronal Cell Death and Developmental Malformations in Reptiles

**DOI:** 10.1093/iob/obab033

**Published:** 2021-12-02

**Authors:** Thomas J Sanger, Laura Harding, Judith Kyrkos, Alexandrea J Turnquist, Lilian Epperlein, Sylvia A Nunez, Dryden Lachance, Seerat Dhindsa, James T Stroud, Raul E Diaz, Beata Czesny

**Affiliations:** Department of Biology, Loyola University Chicago, 1050 Sheridan Rd., Chicago, IL 60660, USA; Department of Biology, Loyola University Chicago, 1050 Sheridan Rd., Chicago, IL 60660, USA; Department of Biology, Loyola University Chicago, 1050 Sheridan Rd., Chicago, IL 60660, USA; Department of Biology, Loyola University Chicago, 1050 Sheridan Rd., Chicago, IL 60660, USA; Department of Biology, Loyola University Chicago, 1050 Sheridan Rd., Chicago, IL 60660, USA; Department of Biology, Loyola University Chicago, 1050 Sheridan Rd., Chicago, IL 60660, USA; Department of Biology, Loyola University Chicago, 1050 Sheridan Rd., Chicago, IL 60660, USA; Department of Biology, Loyola University Chicago, 1050 Sheridan Rd., Chicago, IL 60660, USA; Department of Biology, Washington University in St. Louis, Campus Box 1137. One Brookings Drive St. Louis, MO 63130-4899, USA; Department of Biological Sciences, California State University, Los Angeles, 5151 State University Dr., Los Angeles, CA 90032, USA; Department of Biology, Loyola University Chicago, 1050 Sheridan Rd., Chicago, IL 60660, USA

## Abstract

Every stage of organismal life history is being challenged by global warming. Many species are already experiencing temperatures approaching their physiological limits; this is particularly true for ectothermic species, such as lizards. Embryos are markedly sensitive to thermal insult. Here, we demonstrate that temperatures currently experienced in natural nesting areas can modify gene expression levels and induce neural and craniofacial malformations in embryos of the lizard *Anolis sagrei*. Developmental abnormalities ranged from minor changes in facial structure to significant disruption of anterior face and forebrain. The first several days of postoviposition development are particularly sensitive to this thermal insult. These results raise new concern over the viability of ectothermic species under contemporary climate change. Herein, we propose and test a novel developmental hypothesis that describes the cellular and developmental origins of those malformations: cell death in the developing forebrain and abnormal facial induction due to disrupted Hedgehog signaling. Based on similarities in the embryonic response to thermal stress among distantly related species, we propose that this developmental hypothesis represents a common embryonic response to thermal insult among amniote embryos. Our results emphasize the importance of adopting a broad, multidisciplinary approach that includes both lab and field perspectives when trying to understand the future impacts of anthropogenic change on animal development.

## Introduction

Climate change is pushing many organisms to their thermal limits (Walther et al. 2002; Milligan et al. 2009; Lurgi et al. 2012; Altizer et al. 2013). Ectothermic species, such as lizards, are of special concern as these organisms rely on abiotic conditions to maintain many important physiological processes and behaviors. However, many ectotherms already function at close to their thermal limits (Deutsch et al. 2008), meaning the future viability of such processes under contemporary climate change is unclear (Huey et al. 2009; Sinervo et al. 2010; Carlo et al. 2018). Most overlooked are the threats posed by increased global temperatures that extend beyond free-living stages and into developing embryos (Munday et al. 2008; Pankhurst and Munday 2011; Benard 2015; Levy et al. 2015). Because of their rapid rates of cell division and the numerous signaling pathways involved with tissue differentiation and organ growth, embryos of egg-laying (i.e., oviparous) species, such as many lizards, likely represent one of the most vulnerable life history stages of global ectotherm biodiversity.

Exposure to temperatures above a preferred thermal range (i.e., thermal stress) is a well-known stressor of animal development (Edwards et al. 2003; Edwards 2006; Hansen 2009). However, the context in which embryonic thermal stress has been studied remains limited to few systems, limiting our understanding of whether species respond to thermal stress through the same, similar, or different physiological mechanisms. For example, embryonic thermal stress is a known teratogen and can reduce embryonic survival in mammals, including humans (Webster and Edwards 1984; Edwards 1998; Hansen 2009). Thermal stress may also alter embryonic programming associated with sexual differentiation in turtles (Neuwald and Valenzuela 2011; Valenzuela et al. 2019) and lizards (Holleley et al. 2015, 2016) that have temperature-dependent sex determination. The impending risk of embryonic thermal stress on lizard development has been recognized for the past decade (Warner et al. 2012; Tiatragul et al. 2017; Hall and Warner 2018; Gunderson et al. 2020), but direct study of lizard embryos remains rare. Instead, ecophysiologists have often relied on indirect measurements of embryonic health such as monitoring heart rate (Du and Shine 2008; Du et al. 2010), respirometry studies (Smith, Telemeco, et al. 2015; Hall and Warner 2020), and measuring survivorship and phenotypes of hatchlings (Huang and Pike 2011; Warner et al. 2012; Pearson and Warner 2016; Carlo et al. 2018). A focus on the hatchlings that survive embryonic thermal stress biases later observations to only those embryos that can withstand the challenges experienced during embryonic development. To fully assess the potential impact of global change on lizard embryos, more direct study of the stage- and tissue-specific effects of thermal stress is of pressing importance.

For many lizards, the earliest stages of development, including fertilization, gastrulation, and neurulation, occur within the female (Andrews and Mathies 2000; Sanger, Losos, et al. 2008; Diaz et al. 2017; Griffing et al. 2019). If the thermal range of the female and embryo are similar, thermal stress in these stages may be mitigated by behavioral thermoregulation. However, the majority of lizard species are oviparous and do not exhibit parental care, meaning that later embryonic development is dictated by the abiotic conditions of the nest site. Yet, natural nest sites likely exhibit variable conditions between sun and shade, between early and late breeding seasons, or among populations living in different microclimates. This may be further complicated by extreme climate events, which are increasing in frequency due to global change (Diffenbaugh et al. 2017; Stillman 2019; IPCC 2021). Thus, the ways that thermal stress may affect lizard embryos in the wild are dynamic, increasing the need for well-controlled laboratory experiments that establish the thermal bounds of embryonic development. To determine how climate change may affect lizard development, we investigated the resiliency of developing *Anolis sagrei* embryos to increasing temperatures.

## Amniote craniofacial development

Through our experiments (later), we demonstrate that the developing brain and face of lizard embryos are particularly sensitive to thermal stress that affects the embryo at the time of oviposition. We then develop and test a developmental hypothesis that explains the range of neural and facial malformations observed in embryos exposed to thermal stress, proposing that they may be united under a common mechanism. Although the development of the boney and cartilaginous skull has been described for several squamate species (e.g., Boughner et al. 2007; Hernández-Jaimes et al. 2012; Ollonen et al. 2018; Diaz and Trainor 2019), the details of facial morphogenesis and its molecular patterning have not been previously investigated. Instead, most of our understanding of amniote craniofacial morphogenesis comes from observations of only two model organisms, the chicken (*Gallus*) and mouse (*Mus*). It has long been assumed that the mechanisms of early craniofacial morphogenesis are conserved among vertebrates, but gaps remain in analyses of vertebrate facial development.

The anterior and posterior portions of the skull form through distinct developmental mechanisms (reviewed in Santagati and Rijli 2003; Noden and Trainor 2005; Gross and Hanken 2008). The anterior cranial skeleton, or face, forms exclusively from neural crest cells (Le Lièvre 1978; Noden 1978; Couly et al. 1993; Le Douarin and Dupin 1993; Chai et al. 2000; Jiang et al. 2002). Following neurulation, cranial neural crest cells migrate ventrally from the margin of the neural tube, over the developing brain, until they reach the presumptive oral region. As they proliferate and differentiate into skeletal precursors, they create a space between the oral/supraoral ectoderm and the telencephalon. The telencephalon, therefore, provides both the physical foundation and molecular signals needed for proper facial formation (Marcucio et al. 2005, 2015; Boughner et al. 2008; Hu and Marcucio 2008; Chong et al. 2012). Changes in the size of the telencephalon, such as those observed during the early evolution of humans or in pathological conditions such as holoprosencephaly, will lead to secondary changes in the proportion of the facial skeleton (Weinberg et al. 2009; Marcucio et al. 2011, 2015). Following facial induction, the face forms by the outgrowth and fusion of medial and lateral facial prominences. Different amniote species have distinct arrangements and sizes of these facial prominences (Young et al. 2014; Smith, Percival, et al. 2015), but all undergo this process of outgrowth and fusion.

The developing brain hosts a dynamic series of Hedgehog signaling events that are necessary for proper facial induction (Eberhart et al. 2006; Hu and Marcucio 2008; Chong et al. 2012). First, the secreted signaling molecule, Sonic hedgehog (Shh), is expressed in the diencephalon, which induces a second Shh expression domain in the telencephalon (Marcucio et al. 2005; Hu and Marcucio 2008). This then induces a third zone of Shh expression at the boundary of the oral and supraoral ectoderm. This boundary forms a signaling center that directs the outgrowth of the face (Abzhanov and Tabin 2004) through the proliferation of the underlying undifferentiated neural crest-derived cells (Ahlgren and Bronner-Fraser 1999; Brito et al. 2006). Intracellular Hedgehog signaling, including the Hedgehog receptor Patched1 and the Hedgehog transcriptional activator, Gli1, are observed throughout the undifferentiated neural crest cells (Jeong et al. 2004; Firulli et al. 2014; Wang et al. 2019). Herein, we examine the expression and function of the Hedgehog pathway in craniofacial development to determine whether disruption to this pathway can induce the craniofacial malformations observed under thermal stress.

## Materials and methods

### Husbandry and embryo collection

*Anolis* lizards are an often-used model for studies of ecophysiology and the biological impacts of global change (Hertz and Huey 1981; Gunderson and Leal 2012; Logan et al. 2013; Muñoz et al. 2014; Hall and Warner 2018; Sanger et al. 2018). We collected approximately 185–215 gravid female *A. sagrei* from Coral Gables, FL, in summers of 2016–2018. All females were housed at Loyola University Chicago in accordance with the university's Institutional Animal Care and Use Committee policies (IACUC protocol #1992). Briefly, gravid females are housed in groups of four to six individuals per cage with perches and a pot of moist dirt for them to lay their eggs (Sanger, Hime, et al. 2008). Male lizards were not collected or housed. Female lizards are fed crickets daily and misted five to six times per day with an automated MistKing system (Jungle Hobbies Ltd.). Eggs laid within the first week after transport were not included in this study. We collected eggs daily between 900 and 1000 h and randomly assigned them to an incubation schedule.

To test the resiliency of developing *A. sagrei* embryos to thermal stress, we reared eggs at temperatures ranging from 27°C to 39°C until the completion of morphogenesis at 12 days (Sanger, Losos, et al. 2008). After this time, the major anatomical systems are easily recognizable. This allowed us to assess embryonic phenotypes prior to death and degradation of the embryo. We maintained control eggs at 27°C throughout the duration of our incubation experiments in a VWR personal desktop incubator. Because malformations occur at a low frequency at 27°C, this large sample size allowed us to assess whether malformations that occur at baseline temperatures parallel those that appear under thermal stress or whether different forms of malformation arise under thermal stress. For temperatures with relatively low survivorship, we incubated enough eggs to confidently assess similarities among malformed embryos. Following dissection, embryos were scored for stage based on characteristics of cranial, limb, and scale development, the “normal” staging table of *A. sagrei* (Sanger, Losos, et al. 2008; Sanger et al. 2018) and phenotyped by one or more coauthors. The staging of embryos incubated under different temperatures is variable (Sanger et al. 2018). To the best of our ability, we made stage-matched comparisons among individuals (Sanger stage 11/12) and did not include embryos significantly younger than this in the analysis of malformations. Only living embryos, those with a visible heartbeat, were scored although deceased embryos with severe malformations were observed. To visualize the detailed surface morphology of the embryos, we soaked fixed (overnight in 4% paraformaldehyde) embryos in DAPI (Sigma; 5 μg/mL in phosphate buffered saline [PBS]) for approximately 1 h to visualize the detailed surface morphology of normal and malformed embryos. We photographed DAPI-stained embryos on a Zeiss V16 fluorescent microscope.

### Putative nest temperatures

To create a thermal profile of potential nesting areas, we collected ground temperatures from July 16 to July 23, 2018 at the Fairchild Tropical Botanical Garden (Coral Gables, FL) using Onset HOBO data loggers. Twenty-one data loggers were placed in the ground 3–4 cm deep within 1 m of trees/perches where female *A. sagrei* were observed (female brown anoles do not always perch on trees and are readily observed on rocks, short ground cover, and directly on the ground). We set the data loggers to record temperature every hour and checked their position each morning to assure that they were not disturbed. On the second day of the data collection period, we also collected hourly data on nest site sun exposure using a digital luxmeter (Dr. Meter LX1330B, range: 0–200,000 lux), accurate ±3% at levels <20,000 lux (lux is the unit of luminous intensity). There were no major weather systems (i.e., long periods of cloud cover) during the time of environmental data collection. We conducted a second round of laboratory incubation experiments based on the results of these field data. Following from the natural observations, we performed a second series of incubation experiments to address whether these temperature profiles could induce changes in craniofacial morphology. We modeled two scenarios: a relatively long-term, low-dose thermal stress (36°C for 8 h) and a relatively acute, high heat shock (39°C for 1 h). Both of these scenarios are likely under future warming, but can have the potential to induce different biological responses in the embryo.

### Normal and abnormal craniofacial development in *A. sagrei*

We assayed the expression pattern of Shh using *in situ* hybridization chain reaction following the manufacturer’s protocols (Molecular Instruments; Choi et al. 2016, 2018). To test the function of Shh in *A. sagrei*, we soaked eggs in an increasing concentration of cyclopamine for 1 h (10, 30, 50, 70, and 100 μM; Toronto Research Chemicals; Chen et al. 2002). Cyclopamine disrupts the intracellular signaling cascade of Hedgehog signaling by binding to Smoothened, not by directly altering the expression of Shh (Chen et al. 2002). The concentration series of cyclopamine, originally dissolved in DMSO, was created by diluting the stock solution in PBS. As a control, an equal amount of DMSO was dissolved in PBS as that used in the strongest cyclopamine treatment. To test the stage specificity of Shh function, we applied cyclopamine on the day of oviposition and 48 h later. We incubated eggs from all manipulations at 27°C. To gain a three-dimensional representation of experimental changes in facial morphology, we stained embryos overnight in 1% phosphotungstic acid dissolved in 70% ethanol. Embryos were imaged on a Perkin Elmer Quantum GX2 (80 kV, 80 μA, 18-mm-bore chamber). These scan parameters produce sub-10 μm voxel size. Three-dimensional models were generated in 3D Slicer (Kikinis et al. 2014).

To determine the presence of putative neural crest cells in the facial region, we used Hnk1 immunohistochemistry in whole mount following previously described protocols (Diaz et al. 2019). This antibody has been successfully used to label neural crest cells in other lizard species (Diaz et al. 2019). We diluted HNK1 primary antibody (DSHB, 3H5 IgM) in TN-Block 1:20 and incubated overnight at 4°C on a nutator. On the following day, after 5 × 1 h PBS washes, Goat anti-Mouse (IgM) HRP conjugated secondary antibody (Invitrogen, 626820) was applied as a dilution of 1:500 in TN-Block overnight at 4°C on a nutator. The signal was revealed on the third day after 5 × 1 h PBS washes and subsequent utilization of the DAB Substrate Kit (Roche) for 10–12 min at 4°C on nutator.

### Relative gene expression

To quantify the relative expression of genes potentially involved with neural crest and craniofacial development, we used quantitative reverse transcription polymerase chain reaction (RT-qPCR). As a proxy for undifferentiated neural crest cell number or transcriptional activity, we assayed the expression of the undifferentiated neural crest marker, Sox10 (Sauka-Spengler and Bronner-Fraser 2008; Betancur et al. 2010). We also analyzed three genes associated with Hedgehog signaling: Shh; its receptor, Patched1; and the transcriptional activator of the Hedgehog pathway, Gli1 (Hedgehog pathway reviewed in Briscoe and Thérond 2013; Carballo et al. 2018). We hypothesized that these genes would show modified expression in embryos that experienced thermal stress.

We tested for differences in expression among three incubation treatments on days 0 and 1 of postoviposition embryonic development, respectively. As a control, potentially stressful incubation treatments were compared with embryos reared at constant 27°C for 8 or 24 h. We also collected embryos on the day of oviposition (day 0) after 8 h at 36°C and after a 1-h 39°C heat shock, paralleling conditions that may be observed in nature (see later). To test whether stressful conditions on day 0 translate to lasting changes in gene expression, we returned the thermally stressed eggs to the 27°C incubator for 24 h and collected developing facial tissue on day 1.

We collected day 0 and day 1 embryos from a captive *A. sagrei* colony maintained at Loyola University Chicago (2021). We again dissected them while they were submerged in sterile PBS. We dissected the developing telencephalon and face by making an incision with a 0.125 mm diameter tungsten needle around the posterior margin of the eye. To avoid conflation of experimental differences with differences in embryonic stage, we stage matched embryos. Only Sanger stage 3/4 were analyzed on day 0 and Sanger stage 4/5 analyzed for day 1 (Sanger, Losos, et al. 2008). The dissected tissues were immediately flash frozen in liquid nitrogen and stored at –80°C until the time of RNA extraction. We collected 10 individuals of each stage at each time point and analyzed the eight with the highest RNA yields.

We performed RNA extraction using RNAzol (Molecular Research Center) following manufacturer's protocols, including the suggested 4-Bromoanisole additive. To synthesize complimentary DNA (cDNA) we used the ThermoScientific Maxima First Strand cDNA Synthesis Kit for RT-qPCR using one microgram of total RNA. Final cDNA concentrations were diluted to 200–250 ng/μL for RT-qPCR.

We designed primers for our target genes using publicly available sequences (Table S1). To increase the chances of success, we aligned sequences for multiple squamate species to find regions of sequence conservation and targeted these regions for primer design. Primers were designed to amplify fragments of 80–160 base pairs using the Primer Quest design software (IDT). Only primers with a single peak on the melting curve were used for experimental analysis. We used the PowerUp SYBR Green Master Mix on an Applied Biosystems StepOnePlus (96-well plate) following the manufacturer’s protocols. All reactions were performed in triplicate. We designed RT-qPCR 96-well plates to include amplification of the control and experimental genes on every plate. Pipetting error was evaluated within each technical triplicate within the StepOnePlus software and outliers removed prior to statistical analysis.

We used the delta-delta Ct method (2^–∆∆Ct^) to analyze qPCR data (Livak and Schmittgen 2001). In this method, the expression of the gene of interest was standardized to the expression of the control gene for each sample. The data were natural Log transformed prior to analysis. This means that values below 0 represent lower expression compared with the control and values greater than 0 represent higher expression. We analyzed relative fold differences in expression using a general linear model performed in SPSS version 27 (IBM). To assess whether Hedgehog signaling is modified by thermal stress, we analyzed Shh, Patched1, and Gli1 as dependent variables in a single model. To assess whether neural crest cells are also disrupted by thermal stress, we used Sox10 as a dependent variable in a separate model. Because gene expression may be changing between the developmental stages analyzed on days 0 and 1, we analyzed each day separately. In several cases, the gene of interest failed to amplify even when the control gene amplified within the range of other samples. In these cases, the CT value was omitted from the statistical analysis (late amplification indicates extremely low or undetectable expression for that individual). This only occurred in samples collected from the thermal stress treatments and is, therefore, likely to bias our results toward rejection of the experimental hypotheses.

### Cell proliferation and death

We measured rates of cell proliferation and cell death after 24 and 48 h of incubation within embryos incubated at two temperatures, 27°C and 36°C. To measure rates of cell proliferation we pulse labeled embryos for 2 h with an injection of Edu (Life Technologies). We injected 20 μL of a 20 mg/mL solution into the egg and returned it to the incubators for two hours. Signal recovery followed manufacturer’s protocols. Cell death was assayed using anti-activated caspase-3 (1.5% sheep serum block, Abcam ab13847 1:100; Life Tech. A21428 1:400 secondary) immunofluorescence on the same frozen sections (tissues embedded in Optimal Cutting Temperature Embedding Media, sectioned on Leica CM1850 cryostat). Activated caspase-3 is a highly conserved mediator of programmed cell death in vertebrates (Porter and Jänicke 1999). Three proliferation/death cell counts were taken in a 100 μm × 100 μm box from the ventral telencephalon per individual and averaged before statistical comparison. Because of unequal variances between samples (see later), we compared among samples using a Kruskal–Wallis nonparametric test.

## Results

### Thermal stress induces craniofacial malformations

**Fig. 1:**
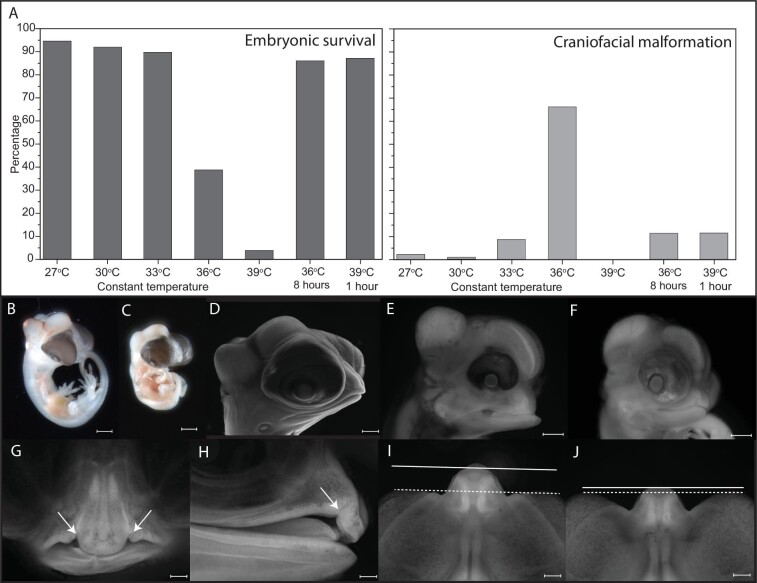
Temperature-induced developmental malformations in brown anole embryos. There is a precipitous decline in embryo survival (**A**, dark gray) and a concomitant increase in the rates of structural malformation (**A**, light gray) between 33°C and 36°C. At 36°C, there is a decrease in body size (**B**, 27°C; **C**, 36°C). Compared with embryos incubated at 27°C **(D)**, we observed brachycephaly **(E, F)**, mandibulary prognathism (E), and facial clefting (F) in embryos raised at elevated temperatures. The temperatures that can induce these malformations are observed in natural nest sites today **(G–J)**. Incubation regimes based on field observations can induce bilateral (G) or unilateral (H) facial clefting after only 8 h at 36°C. Compared with embryos incubated at temperatures similar to shady nest sites (J), embryos that experience only one hour heat shock of 39°C exhibit noticeable changes in facial length (J; dashed line serves as a reference at eye level, solid line highlights the tip of the snout). Each scale bar is 1 mm.

Our first incubation experiment, under stable incubation conditions, revealed a critical thermal window between 33°C and 36°C across which we observe a dramatic reduction in survivorship and a concomitant increase in structural defects (Fig. 1A; Table 1). At an incubation temperature of 27°C, we recorded 95% survival (total *N* = 259) and a baseline malformation rate of 1.6% of surviving embryos. At 27°C, we observed only four malformed embryos: one with dramatic reduction in body size and spinal contortions, one conjoined twin, and two severely malformed embryos with unrecognizable morphology. Increased temperature yielded a dramatic decrease in body size (Fig. 1B and C; Sanger et al. 2018). At incubation temperatures of 36°C, only 38% of embryos survive (total *N* = 106). Of the surviving embryos, 66% exhibited structural defects such as abdominal edema, organ exstrophy, or vertebral malformations. Of the malformed embryos, 65% had anterior craniofacial malformations, including brachycephaly, mandibular prognathism, diprosopus, and clefting of the facial skeleton (Fig. 1D–F; Fig. S1; Table 1).

**Table 1:** Summary of egg incubation experiments

Incubation conditions	Total eggs incubated	Percentage survived to 12 days	Percentage malformed	Observed craniofacial malformation classifications
Temperature resiliency				
27°C constant	259	95%	1.60%	
30°C constant	51	92%	0%	
33°C constant	87	90%	8%	Diprosopus^*^, brachycephaly, mandibular prognathism
36°C constant	106	39%	66%	Brachycephaly, mandibular prognathism, unilateral clefting, bilateral clefting
39°C constant	94	4%	—	
Ecological context				
36°C for 8 h, 27°C until 12 days	65	86%	11%	Brachycephaly, unilateral clefting, bilateral clefting
39°C for 1 h, 27°C until 12 days	60	87%	11%	Brachycephaly, changes in facial proportion
Stage specificity				
Females maintained at 36°C, eggs at 27°C until 12 days	88	60%	8%	Loss of midline facial tissue^*^, brachycephaly, mandibular prognathism
36°C for 48 h, 27°C to 12 days	54	84%	11%	Brachycephaly, mandibular prognathism, unilateral clefting, bilateral clefting
27°C for 48 h, 36°C to 12 days	51	51%	0%	
**Total number of eggs**	**915**			

* See Figs. S1 and S5 for further details.

### High ground temperatures can induce structural malformations

**Fig. 2:**
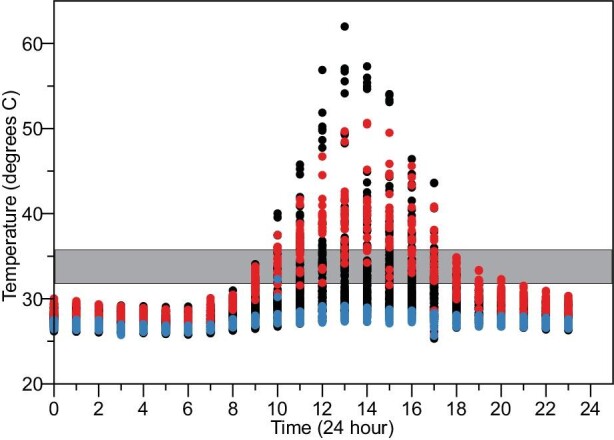
Temperature profiles of putative *A. sagrei* nest sites. Nest sites with high sun exposure (three highest Lux sites in red) exhibit considerably more variation in temperature than those in the shade (three lowest Lux sites in blue; black dots represent data from remaining 15 data loggers). Gray box represents the critical thermal window of *A. sagrei* embryos revealed by our incubation experiments. Time 0 represents 12:00 am.

Female brown anoles typically nest in loose soil at a depth of 3–4 cm within 1 m of vegetation (Hall and Warner 2018; Tiatragul et al. 2019). To determine how our baseline measures of thermal sensitivity compare with the temperature range of potential nest sites, we collected ground temperatures, 3–4 cm deep, across the full spectrum of microhabitats. There was wide variability in the temperature profile of putative nest sites, correlating with their degree of sun exposure (Fig. 2; Figs. S2 and S3; Table S2). Nest sites with low sun exposure were relatively stable throughout the day (Fig. 2; Figs. S2 and S3; Table S2; minimum: 26°C; maximum: 32°C; SD < 1°C). In contrast, nest sites with high sun exposure showed considerable variation in daytime temperature (Fig. 2; Figs. S2 and S3; Table S2; minimum: 25°C; maximum: 62°C; SD > 6°C). Putative nest sites with low sun exposure did not reach the critical thermal range of brown anole embryos, while those with high to moderate sun exposure were higher than 36°C for 6–8 h per day (Table S2).

Based on our observed temperatures of nesting sites in the wild, we simulated short-term heat stress in two ways. First, we exposed *A. sagrei* embryos on the day of oviposition to 36°C for 8 h before returning them to the 27°C incubator until day 12. Of these embryos, 86% survived (*N* = 65), but 11% showed craniofacial malformations, including a shortened or downturned snout, unilateral, or bilateral clefting (Fig. 1G and H). Second, we exposed eggs to a 1 h 39°C thermal shock. Survival in these embryos was high (*N* = 60, 87% survival), but 11% had changes in facial proportion, most commonly brachycephaly and mandibular prognathism (Fig. 1I and J). Our incubation experiments demonstrate that elevated levels of craniofacial malformation can be induced at ground temperatures that are observed today.

### Normal and abnormal craniofacial development in *A. sagrei*

**Fig. 3:**
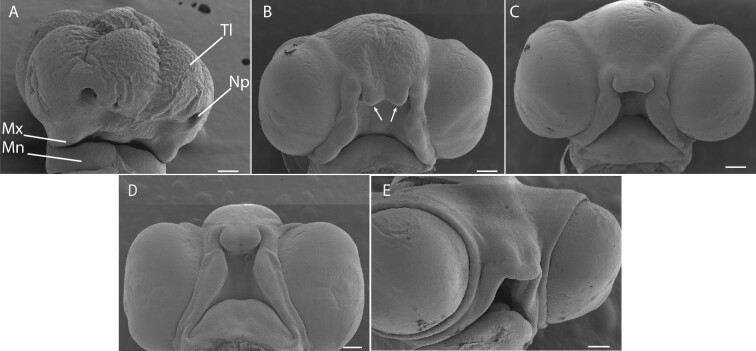
Craniofacial development of *A. sagrei*. The timing of craniofacial development in *A. sagrei* is consistent with the time that Shh may be disrupted by thermal stress. At oviposition **(A)**, maxillary (Mx) and mandibulary (Mn) processes are present. The bifurcated midline facial prominences do not appear until day 3 (**B**, arrows). Over the next several days (**C**, days 6–7; **D**, days 8–9), the facial prominences grow and begin to fuse with the midline facial prominences. By days 11–12, a recognizable, forward-facing snout is present. (Tl, telencephalon; Np, nasal pit; A, B scale bar = 1 mm; C, D, E scale bar = 2 mm).

The stages of facial development that unfold over the first 48 h following oviposition are consistent with those where perturbation of Hedgehog signaling could occur (Fig. 3A–E). At oviposition, the maxillary (Mx) and mandibulary (Mn) processes of *A. sagrei* are visible, but paired frontonasal processes do not appear until postoviposition day 3 (Fig. 3B). By days 11–12, the facial processes have elongated and fused to form a recognizable face with a smooth oral boundary (Fig. 3E).

**Fig. 4:**
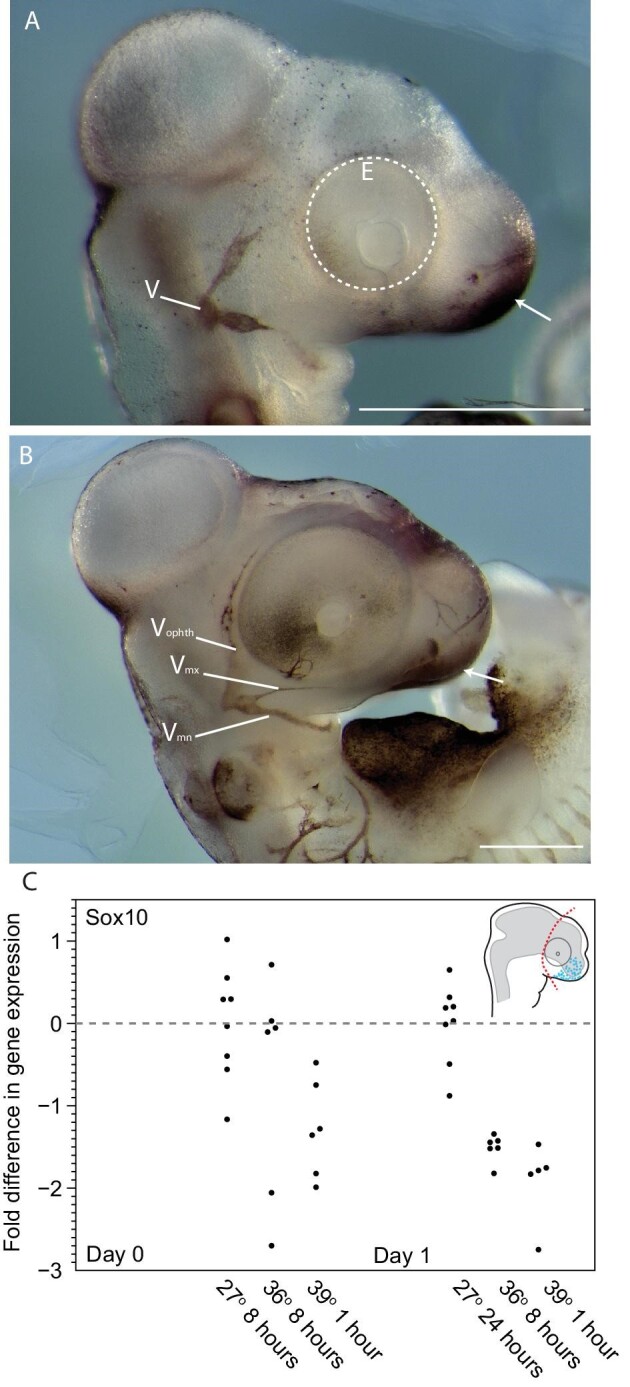
Undifferentiated neural crest cells in early *Anolis* embryos. **(A)** At oviposition, HNK1 positive cells (dark staining) are observed in the most anterior portion of the presumptive facial region (arrow) and within the developing trigeminal nerve (cranial nerve V; V). This pattern is maintained throughout the first 48 h of postoviposition development **(B)**. At this time, HNK1 positive cells remain in the presumptive facial region, although the trigeminal nerve is further developed (V_max_ = maxillary branch of V; V_mand_ = mandibulary branch of V; and V_opth_ = ophthalmic branch of V). **(C)** Our RT-qPCR experiment of the undifferentiated neural crest marker, Sox10, reveals that elevated incubation temperatures lead to reduced transcriptional activity or a reduced number of neural crest cells. Although there are not statistically significant differences in mean expression level on the day of oviposition, lower levels of Sox10 are observed in some individuals immediately following thermal stress (i.e., Day 0). These differences become exacerbated among all individuals the following day (i.e., Day 1), even though the thermal insult was already removed. Statistical results of the RT-qPCR experiment are summarized in Table 2.

HNK1 immunohistochemistry revealed HNK1 positive cells, undifferentiated neural crest cells, populating the presumptive facial area and labeling the cranial nerves during the first 1–2 days of postoviposition development (Fig. 4A and B). As a proxy for cranial neural crest number and/or cell transcriptional activity, we used RT-qPCR to quantify the expression of Sox10, a marker of undifferentiated cranial neural crest cells (Sauka-Spengler and Bronner-Fraser 2008; Betancur et al. 2010), among stage-matched embryos experiencing different incubation conditions. The univariate linear model recovered a marginally insignificant difference in Sox10 expression on day 0 (model *P* = 0.057; Fig. 4C; Table 2). There was a highly significant difference among treatments in Sox10 expression on day 1 (*P* < 0.01; Fig. 4C; Table 2). Tukey post-hoc tests recovered significant differences between both stressful incubation treatments and the constant 27°C control. This result suggests that there are fewer or less transcriptionally active cranial neural crest cells in embryos that have experienced thermal stress.

**Table 2:** Results of quantitative gene expression analyses

Neural crest marker			
		**Day 0**	**Day 1**
	Dependent variable	Sox10	Sox10
	Model significance	0.057	***P* < 0.001**
	*F*	3.412	43.511
	Degrees of freedom	2	2
**Hedgehog signaling**			
	Dependent variables	Shh	Shh
		Patched1	Patched1
		Gli1	Gli1
	Wilks' lambda significance	***P* < 0.001**	**0.006**
	*F*	33.700	5.701
	Error degrees of freedom	18	19
	Hypothesis degrees of freedom	3	3
	Treatment: Shh		
	Significance	0.113	**0.011**
	*F*	2	2
	Degrees of freedom	2.438	5.655
	Treatment: Patched1		
	Significance	**0.005**	0.06
	*F*	2	2
	Degrees of freedom	7.022	3.234
	Treatment: Patched1		
	Significance	***P* < 0.001**	0.100
	*F*	2	2
	Degrees of freedom	21.390	2.569

**Fig. 5:**
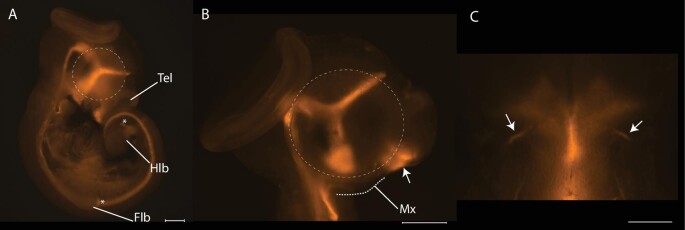
The expression and function of Shh in *A. sagrei*. At oviposition, Shh is expressed within the ventral telencephalon (**A**; **B**, arrow). Shh is also expressed within the limb bud (Flb, forelimb; Hlb, hindlimb) and notochord at this time (A). Approximately 36–48 h after oviposition **(C)**, two transitory domains of Shh mark the future position of facial prominences. Panel (C) shows the superior aspect of the oral epithelium prior to the formation of the midline facial prominences. Scale bars equal 1 mm.

**Fig. 6:**
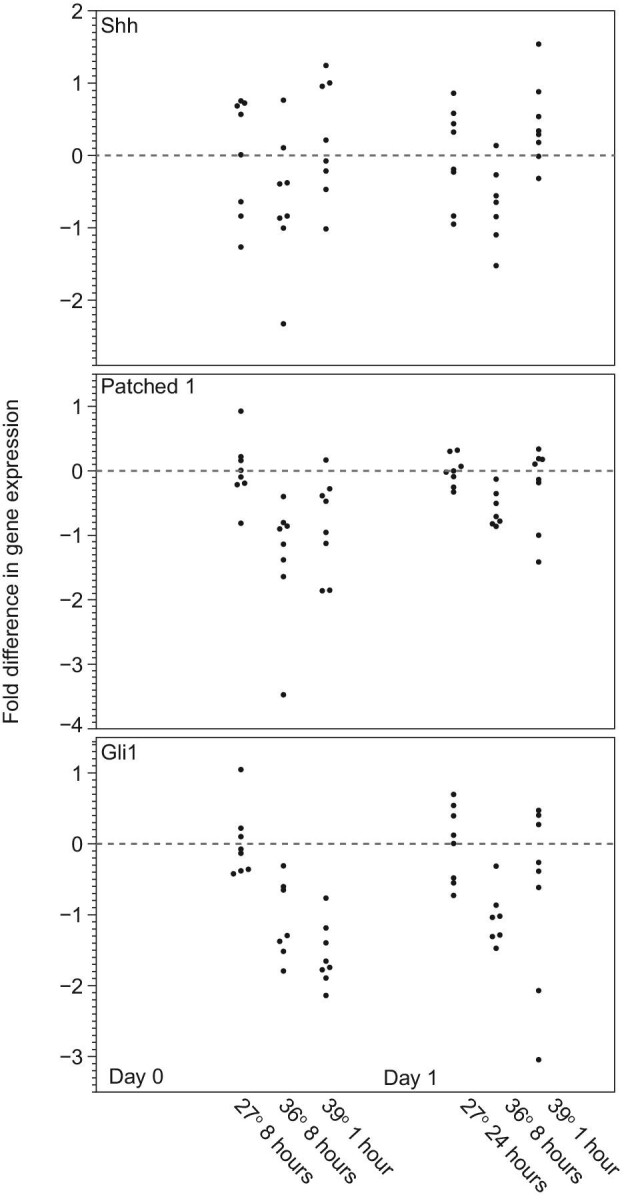
RT-qPCR results for members of the Hedgehog signaling pathway. Quantification of gene expression of the Hedgehog pathway in embryos incubated under different thermal profiles reveals modification in signaling in embryos incubated under elevated temperatures. The most notable changes in Hedgehog signaling are observed on Day 0, immediately after thermal stress has impacted the embryo. In these embryos, there are pronounced differences in the expression levels of Patched1 and Gli1. On Day 1, after the period that thermal stress has been removed, there are no statistical differences in Hedgehog gene expression between embryos incubated at relatively low and high temperatures, although there are differences between thermal stress treatments. Statistical results are summarized in Table 2.

At the time of oviposition, Shh is expressed in the ventral telencephalon and limbs (Fig. 5A and B). Limb development is not disrupted in thermally stressed embryos, indicating that the observed phenotypes are not due to Shh instability. Approximately 48 h after oviposition, two transitory domains of Shh expression within the oral epithelium mark the position of the paired frontonasal processes (Fig. 5C). We used RT-qPCR to quantify Hedgehog signaling in embryos incubated under different conditions. Although the details vary between day 0 and 1, our analysis shows significant differences in Hedgehog signaling in the developing faces of embryos experiencing thermal stress (Fig. 6; Table 2). On the day of oviposition, the expression of Patched1 and Gli1 exhibits significant differences among incubation treatments (Table 2). Tukey post-hoc tests confirm that both the relatively high short-term heat shock and prolonged thermal stress induced significant changes in Hedgehog signaling compared with the control (Table 2). On day 1, Hedgehog signaling is significantly different among treatments, although Tukey post-hoc tests reveal that none of the stressful incubation treatments are significantly different than the 27°C control. The model's significance is the result of differences in expression between the two stressful incubation conditions.

**Fig. 7:**
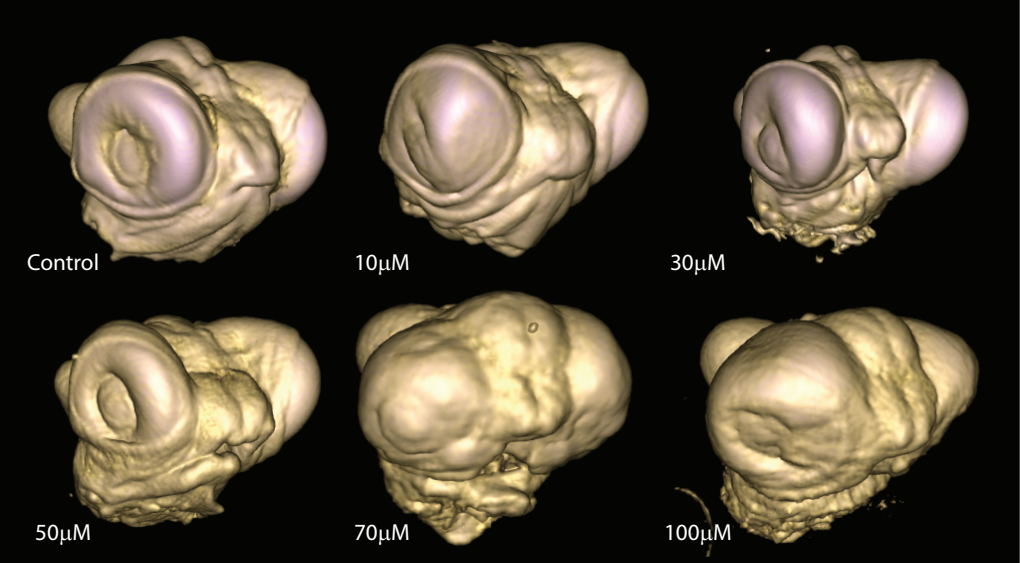
Functional analysis of Shh in the developing head of *A. sagrei*. Increasing concentrations of cyclopamine lead to a reduction in midline facial tissue and shortening of the face. At 50 μM concentrations, clefting between the maxillary and midline facial prominences is visible in some embryos. Concentrations above 70 μM lead to the complete ablation of midline facial tissue (pers. obs.). The small anteriorly facing projections between the eyes are the remainder of the olfactory processes with a thin ectodermal covering.

We tested the function of Shh in the developing lizard face by treating embryos on the day of oviposition with a concentration series of the hedgehog antagonist cyclopamine. Increasing concentrations of cyclopamine led to a shorter face and reduction of midline facial tissue, although the maxillary processes continue to extend toward the midline (Fig. 7). The effects of Shh disruption occur rapidly. Three days after cyclopamine treatment, midline facial processes fail to form under high cyclopamine concentrations (Fig. S4A). Applying cyclopamine 48 h after oviposition leads to minor changes in facial proportion, but structural malformations, including clefting and brachycephaly, were not observed (Fig. S4B). This experiment indicates that disruption of Shh during the first 2 days of postoviposition development produces phenotypes consistent with those induced under thermal stress.

**Fig. 8:**
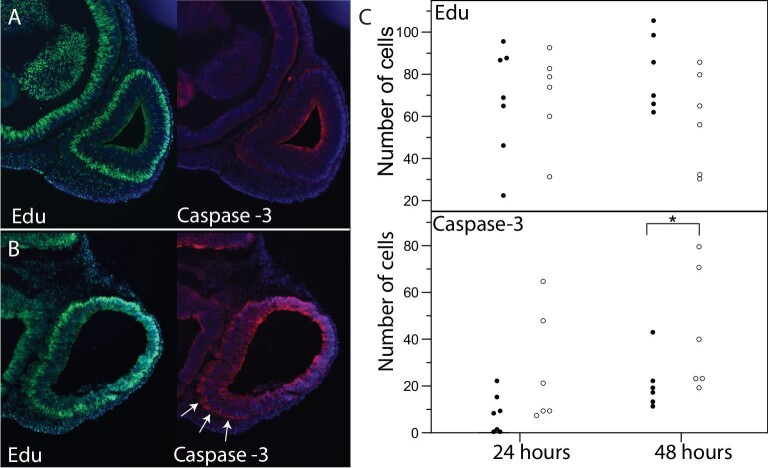
Proliferation and cell death in thermally stressed *A. sagrei* embryos. Embryos incubated at 27°C **(A)** show widespread proliferation and low levels of cell death in the telencephalon. In contrast, embryos incubated at 36°C **(B)** show a wide domain of cell death in the ventral telencephalon (arrows). Quantification of these patterns (**C**: closed circles represent individuals incubated at 27°C and open circles 36°C) demonstrates that the number of apoptotic cells increases over the first 48 h of development (*N* = 6 for each time and treatment; ^*^P < 0.05 Kruskal–Wallis nonparametric test).

We next examined whether elevated incubation temperatures changed the pattern of cell proliferation and cell death in the ventral telencephalon. There was no difference in cell proliferation, the number of Edu positive cells, between embryos reared at 27°C and 36°C (Fig. 8). In contrast, several embryos from each treatment (two embryos from 24 h at 36^o^C and two embryos from 48 h at 36^o^C) of embryos reared at 36°C exhibited an expanded region of cell death in the area coincident with the Shh expressing domain of the telencephalon (Fig. 8). Because this region was not distinct in every embryo, we quantified activated caspase-3 positive cells in the ventral telencephalon. This revealed that thermally stressed embryos reach higher levels of cell death and that there is considerable variation in the amount of cell death among embryos. We did not observe putative neural crest cells, mesenchymal cells ventral to the telencephalon, positive for anti-activated caspase-3. Although we did not observe differences in the average number of activated caspase-3 positive cells, the number of individuals we observe with increased rates of cell death is consistent with the relatively rare onset of craniofacial malformations in thermally stressed *A. sagrei* embryos. The range of variation in cell death within thermally stressed embryos could potentially explain the variation of Hedgehog signaling and, in turn, the range of neural and craniofacial phenotypes we observed under elevated incubation conditions (Fig. 1B–J).

**Fig. 9:**
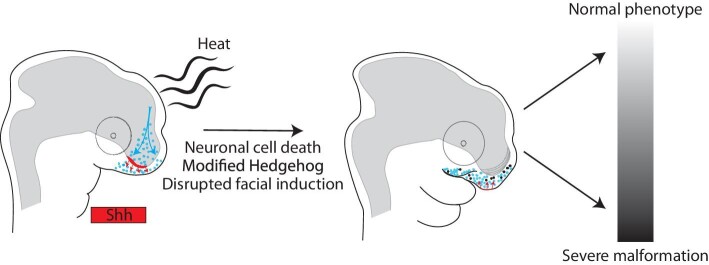
A developmental model of embryonic thermal stress. Our developmental model predicts that thermal stress leads to cell death in the developing telencephalon, specifically in the region associated with Shh secretion. Modified levels of Hedgehog signaling then disrupt facial induction. Individual variation in the amount of cell death leads to varying levels of Shh secretion and the spectrum of phenotypes, from normal to severe, observed in our incubation experiments.

We further tested the stage specificity of the thermal sensitivity of *A. sagrei* embryos. Because the earliest stages of anole development occur within the female (Sanger, Losos, et al. 2008), we collected eggs laid by gravid females maintained at 36°C and then incubated them at 27°C until postoviposition day 12 (Fig. S5). This led to a moderate increase in facial malformation above baseline rates (*N* = 88 eggs, 8% malformed, Fig. S5). In contrast, 11% (*N* = 54) of embryos exhibited malformations when incubated at elevated temperatures for the first 48 h postoviposition before being returned to 27°C incubators until postoviposition day 12. Incubating eggs at elevated temperatures after the first 48 h postoviposition dramatically reduced survival (51%, *N* = 51), but did not induce craniofacial malformations (Fig. S5). In addition to confirming the thermal sensitivity of the first 48 h postoviposition, these results suggest that the induction of structural malformations and embryonic survival may manifest through distinct, stage-specific mechanisms.

## Discussion

In the face of contemporary climate change, many organisms are threatened with exposure to temperatures that exceed their physiological limits. Much research has focused on how such climatic conditions pose risks to adult ecology and physiology (Deutsch et al. 2008; Huey et al. 2009; Sinervo et al. 2010). However, several ecological studies have already observed that natural reptilian nest sites are currently experiencing conditions within the critical temperature range raising new concern about the viability of ectothermic embryos (Neuwald and Valenzuela 2011; Holleley et al. 2016; Hall and Warner 2018; Tiatragul et al. 2019; Bock et al. 2020). Here, we identify that contemporary climate change likely presents a major, yet underappreciated, threat to biodiversity at the level of an embryo: cell death in the developing forebrain and the emergence of craniofacial malformations. Yet, one of the greatest challenges we will have to overcome is to address how we study the development of rare phenotypes in wildlife that are not closely related to the commonly used vertebrate model organisms, such as squamates and non-avian archosaurs. Developmental malformations tend to be relatively rare events and large numbers of embryos are required to understand their embryological origins and the mechanisms by which they are induced. Prior studies have reported a reduced rate of hatching from reptile eggs incubated at elevated temperatures, but did not explore the underlying mechanisms for why those embryos failed to hatch or the developmental stages where embryos died (e.g., Pearson and Warner 2016; Carlo et al. 2018; Hall and Warner 2018). Although we have yet to assess hatchling phenotypes, we have observed craniofacial anomalies in 1 in every 10–30 embryos reared under different thermal stress profiles. There is potential that these malformations will be more readily realized in the wild as a greater proportion of nest sites move into the critical thermal range during the peak summer months.

In addition to gradual temperature increases, extreme climate events, such as prolonged heat waves and acute temperature spikes, are predicted to increase in frequency and severity in coming decades (Diffenbaugh et al. 2017; Stillman 2019; IPCC 2021), raising the likelihood that a higher percentage of lizard nest sites will experience conditions within in the critical thermal range of embryonic development. Although extreme climate events are often only brief, here we show that short exposure to extreme heat can have substantial effects on embryonic development, even after the period of stress has subsided. Due to the unpredictability of extreme climate events, it remains unclear whether female lizards will be able to anticipate such extreme thermal conditions and so mitigate the effects of climate change on embryos by selecting more thermally sheltered nest sites, suggesting such extreme climate events may represent an emerging threat to ectotherm development, fitness, and survival.

The thermal limits of most squamate embryos remains unknown, yet many lizards lay eggs at the early stages of facial morphogenesis or earlier developmental stages (Andrews and Mathies 2000; Sanger, Losos, et al. 2008; Diaz et al. 2019; Griffing et al. 2019). This raises the possibility that neural and craniofacial malformations may be induced across additional species. Although detailed anatomical descriptions and incubation conditions were not always provided, dramatic or subtle changes in brain and/or facial proportion have also been reported in a range of other reptiles (Yntema 1960; Mulder 1995; Braña and Ji 2000; Amiel et al. 2011; Simoniello et al. 2016) and within the chick developmental model (Hutson et al. 2017) when exposed to elevated incubation temperatures. The malformations observed herein are also similar to the range of phenotypes observed in human holoprosencephaly, which may arise from genetic or environmental insult, including mutations in the Hedgehog pathway and hyperthermia, respectively (Shiota and Opitz 1982; Shiota 1988; Edwards et al. 2003; Cordero et al. 2004; Petryk et al. 2015). Among mammals other than humans, thermal stress is associated with neural tube defects, reduction in brain size, and changes in facial proportion (Pleet et al. 1981; Walsh et al. 1987; Edwards 1998, 2006). These observations suggest that there may be a common thermal stress response among amniote embryos, regardless of the precise temperature where the stress response is induced or whether the species are ectothermic or endothermic.

Changes may not always be dramatic disruption in facial morphology. Instead, alterations may be in the size, shape, or function of the face or forebrain. Because the telencephalon gives rise to the cerebrum, the part of the brain responsible for higher level thinking associated with complex behaviors, individuals that appear to have normal cranial morphology may still be affected by thermal stress in less obvious ways. For example, the mechanisms described herein may explain deficits in learning ability in the gecko *Amalosia lesueurii* (Dayananda and Webb 2017; Abayarathna and Webb 2020) and changes in social behavior in the agamid lizard *Pogona vitticeps* (Siviter et al. 2017, 2019) when eggs were incubated at elevated temperatures. Likewise, the volume of the telencephalon became smaller in eggs reared at relatively elevated temperatures for the skink, *Bassiana duperreyi* (Amiel et al. 2011). Cross-disciplinary analyses of development, neurobiology, and behavior may be necessary for future investigation as integrative biologists try to narrow in on the long-term effects of embryonic thermal stress.

### Squamate craniofacial development and the origin of structural malformations

We performed the first molecular analysis of squamate craniofacial development, lending new support to the conserved nature of the core Hedgehog facial induction mechanism. We performed this analysis within the context of temperature-induced neural and craniofacial malformations. We demonstrated that Hedgehog signaling is directly affected in embryos experiencing thermal stress (day 0). Based on the totality of our results, we propose that neural and craniofacial defects are mechanistically linked in *A. sagrei*: cell death in the developing telencephalon disrupts normal Hedgehog signaling and the induction of the face (Fig. 9). Similar observations have been made for craniofacial defects in models of human disease (Demyer et al. 1964; Cordero et al. 2004; Marcucio et al. 2011; Petryk et al. 2015).

Our RT-qPCR experiment revealed that the strongest changes in gene expression were in genes expressed in the undifferentiated neural crest cells (Patched1, Gli1, and Sox10), not the developing telencephalon (Shh). This raises the possibility that the effect of thermal stress is directly on the neural crest cells, not the developing brain. We find that this is less plausible than the single mechanism we propose. Foremost, many of the most extremely malformed embryos exhibited both neural and craniofacial malformations, such as the embryo depicted in Fig. S2 where the entirety of the midline neural and facial tissue is missing. We have also observed individuals with increased cell death in the telencephalon associated with increasing thermal insult. We also do not see disruption of other neural crest derived tissues, such as skin melanocytes and teeth (Sanger pers. obs.). We cannot rule out that our RT-qPCR results are biased by the larger domain of Shh expression between the developing midbrain and forebrain, which may overwhelm relatively subtle changes in ventral Shh expression that induce facial development (Fig. 5B). Future research will need to further test this possibility with more precise microdissection of the tissue responsible for facial induction and or precise manipulation of the cells in the ventral telencephalon.

Among vertebrates, Shh is necessary for the proper survival and proliferation of the cranial neural crest cells, the precursors to the facial skeleton (Ahlgren and Bronner-Fraser 1999; Marcucio et al. 2005; Hu and Marcucio 2008; Chong et al. 2012). Shh is expressed in a dynamic pattern, first in the diencephalon, then in the telencephalon, and finally in the oral ectoderm (Hu et al. 2003; Marcucio et al. 2005, 2011; Hu and Marcucio 2008). In anoles, disruption of Hedgehog signaling interferes with the formation of the tissue derived from the midline facial prominences, although maxillary derivatives appear to form normally (Fig. 7; Fig. S4). The range of phenotypes observed in our experiments would, therefore, be the result of embryo-specific amounts of cell death altering the levels of Shh in subtle ways. Further research is needed to understand the axis that relates subtle differences in Shh in the telencephalon to neural crest cell proliferation and the resultant downstream phenotypes. Within chickens and mammals, the Shh dose response–phenotype relationship is nonlinear (Young et al. 2010). Small changes in Shh expression may initially lead to dramatic changes in phenotype, after which the relative effect of expression differences dissipates (Young et al. 2010). In this example, expression of Shh at relatively high and relatively low levels led to a shortened upper jaw, but potentially through different cellular mechanisms. If *Anolis* embryos exhibiting diprosopus follow this same pattern, their phenotype may arise because of unique patterns of cell death disrupting normal facial morphogenesis, not simply because of a change in Shh expression level. While we believe that we have demonstrated well that the face and brain are sensitive to thermal insult, there are a range of studies necessary to more thoroughly understand normal craniofacial development in squamates and the ways environmental insult translates to changes in normal development.

### Integration of perspectives is critical to understand the impacts of anthropogenic change

Gaining understanding about the ways that anthropogenic change will impact biological diversity will require a plurality of approaches (Gilbert 2021; Sanger 2021). Comparative, observational, and experimental approaches need to be synthesized to develop a well-rounded understanding of how external stresses strain intrinsic biological processes. Further complicating this challenge, different life stages—adult, juvenile, and different developmental stages—will likely respond to environmental challenges in different ways. Due to the rapid rates of cell division and active processes of tissue differentiation, embryonic life is likely highly sensitive to environmentally induced perturbation. Although the methods and perspectives of developmental biology have not been widely adopted for the study of anthropogenic change, this synthesis has the potential to shed important light on the mechanisms by which an embryo falls into states of dysfunction (Sanger 2021). There are, however, important technical differences between the approaches developmental biologists use and current trends in studies of squamate physiology.

Recent studies on the embryonic physiology of squamates have focused heavily on incubation models that precisely reflect variation in the daily temperature cycles that nest sites experience in the wild (reviewed in Hall and Sun 2021; Taylor et al. 2021). These studies often assess rates of survival at hatching and/or the phenotypes of post-hatching individuals. Physiological studies have often focused on noninvasive techniques such as measuring embryonic heart rates (Du and Shine 2008; Du et al. 2010) or rates of embryonic respiration (Smith, Telemeco, et al. 2015; Hall and Warner 2020). These studies do not readily address the mechanisms that cause certain embryos to die and allow others to survive the same stress. Although several studies have examined changes in embryonic gene expression associated with increasing incubation temperatures (Gao et al. 2014; Takii et al. 2017; Feiner et al. 2018), they have not tried to precisely tease apart the tissue- and stage-specific effects of thermal stress and their relationships with phenotypic modifications. These later questions are where a more formal developmental perspective can provide unique insights.

Developmental biology, evo-devo, and eco-devo have a deep experimental repertoire that is already well tooled to uncover the most sensitive stages, tissues, and signaling pathways in embryos experiencing environmental stress (Sanger 2021). This toolkit provides developmental biologists with the ability to functionally validate mechanistic hypotheses of embryonic dysfunction. Using manipulations such as the application of small molecule inhibitors (as we have) or transgenic technologies, developmental biologists can test whether the disruption of certain signaling pathways creates phenotypes that parallel those observed in stressed embryos. A potential criticism of these approaches is that these experiments do not readily reflect what is observed in the wild. However, manipulating gene expression to supra- or subphysiological levels is one of the core techniques of developmental biology. For example, knockout mice and CRISPR mutants remove pieces of DNA from an organism's genome to assess the function of that gene during development. These techniques have revolutionized our understanding of the genotype-to-phenotype relationship, even though these organisms have no place outside of the laboratory. Rather than dismissing the potential importance of these manipulative experiments, we propose a more thorough integration of environmental variation into this experimental repertoire. Integrative developmental biology has the potential to meet the emerging needs of the 21st century (Gilbert 2021; Sanger 2021).

While we agree that caution needs to be applied when extrapolating from laboratory experiments to the field, we also feel strongly that elucidating the mechanisms of embryonic dysfunction is critically important. This is particularly true for relatively rare events, such as developmental malformations, which are difficult to observe in wild populations, but may have significant impact on populations over multiple generations. Understanding mechanism opens the door for predictive genetic analyses about the ways that future selection may affect biological processes and, in select cases, the application of therapeutics targeting the most responsive or sensitive pathways. Furthermore, pushing our experiments beyond the limits of what is observed today may help predict what organisms will experience under future climactic conditions or during extreme climactic events. Ultimately, there is much to gain by embracing the developmental and experimental perspectives in studies of squamate thermal stress.

## Conclusions

Further understanding the ways thermal stress will impact individual fitness will require integrative analyses spanning the whole of organismal biology. There is a growing literature on adult thermal physiology of lizards in the context of global change (Deutsch et al. 2008; Huey et al. 2009; Sinervo et al. 2010; Logan et al. 2013; Muñoz et al. 2014), but greater investment must also be made toward understanding the stage-, tissue-, and temperature-specific effects of thermal stress on lizard embryos. Our results indicate that there is a correlation between the severity of the thermal insult and the resultant phenotypes; a low intensity thermal insult over a long period of time may induce a similar result as a short, but severe shock to the developing embryo (Walsh et al. 1987; Edwards 2006). Although severely malformed lizards will likely have low fitness and may not reach hatching, we do not yet know whether seemingly unaffected lizards will have deficits in growth, physiology, behavior, or morphology after hatching. We suggest that future studies of normal and abnormal embryonic development incorporate deeper investigation of the Hedgehog pathway and how the perturbation of this pathway translates to changes in brain and facial development. With this synthesis, we can develop a better understanding of the effects of climate change on biodiversity.

## Supplementary Material

obab033_Supplemental_File

## Data Availability

All of the data needed to assess and replicate this research are contained within the manuscript and/or the Supplementary Material.
