# Supplementary material for: Environmental Thermal Stress Induces Neuronal Cell Death and Developmental Malformations in Reptiles
Source: Integr Org Biol. 2021 Dec 2;3(1):obab033. doi: 10.1093/iob/obab033 (PMC8643577; doi:10.1093/iob/obab033)

Supplementary Materials for

Environmental Thermal Stress Induces Neuronal Cell Death and Developmental Malformations in Reptiles

Supplementary Tables

Table S1: Primer sequences used for RT-qPCR
Table S2: Sun exposure and temperature data
Table S3: Tukey post hoc tests for differences from 27° control

Supplementary Figures

Figure S1: Rare, but extreme examples of observed craniofacial malformations
Figure S2: Positive correlation between maximum ground temperature and sun exposure
Figure S3: Examples of putative nest sites
Figure S4: Stage-specific effects of Shh on *A. sagrei* facial development
Figure S5: Stage specificity of induction of craniofacial malformations

Table S1: RT-qPCR primer sequences

**Hedgehog signaling**	
Shh - NCBI: KX778832	
Shh reverse	5' AAGTTAACCCAAGCGGTGCC 3'
Shh forward	5' ATCACCACCTCGGATCGGG 3'
Patched 1 - NCBI: KX778834	
Ptch reverse	5' GCAAACCTCACAGTGCTTGG 3'
Ptch forward	5' TTGGGTGCTTTGAACGGACT 3'
Gli1 - NCBI: KX778833	
Gli1 reverse	5' CGAACTAGGGTCGGTGTAGC 3'
Gli1 forward	5' CAGAACCGGACACACTCCAA 3'
**Neural crest marker**	
Sox10 - NCBI: XM_003227146	
Sox10 Reverse	5' ATGGCAGTGTATAAGGGCCG 3'
Sox10 Forward	5' TAGTCTGCCCCACTATGGCT 3'
**Control gene**	
Beta-actin- NCBI: XM_008123875	
ActB Forward	5' TGGCTCCCAGCACAATGAAA 3'
ACTB Reverse	5' GATGGAAGGTCCGGATTCGT 3'

Table S2: Sun exposure and temperature data

Hobo	LUX	High temp	Low temp	SD temps	Range	Average hours above 36°C	Maximum consecutive hours above 36°C
1	62511.2	40.28	27.28	3.56	13.00	3.86	6
2	68469	42.30	27.45	4.16	14.84	4.86	8
3	35701.9	40.75	26.85	3.59	13.90	3.29	7
4	60315.6	61.99	26.85	5.46	35.14	4.71	6
5	52128	57.31	25.31	8.75	32.00	6.71	8
6	26461	37.19	26.64	2.46	10.55	0.57	3
7	73427	50.66	25.78	6.26	24.88	6.14	7
8	27246.1	39.77	27.15	2.38	12.61	0.86	3
9	12534.5	34.06	25.48	1.49	8.58	0.00	0
10	25750.1	40.32	26.21	2.52	14.11	0.14	1
11	35628	36.38	26.38	2.75	10.00	0.43	1
12	14341	34.10	27.15	2.05	6.95	0.00	0
13	7058	33.46	27.93	0.98	5.53	0.00	0
14	5648	32.47	25.99	1.50	6.48	0.00	0
15	2762.5	29.56	25.87	0.83	3.69	0.00	0
16	5075.1	31.66	26.64	1.27	5.02	0.00	0
7	11618.2	32.60	27.11	0.78	5.49	0.00	0
18	3449.6	30.03	26.04	0.88	3.99	0.00	0
19	2015.4	29.17	25.65	0.88	3.52	0.00	0
20	189.8	32.30	26.21	0.65	6.09	0.00	0
21	666.8	30.20	25.74	0.59	4.46	0.00	0

Table S3: Tukey post hoc tests for differences from 27° control

	Day 0		Day 1	
**Shh**	36° 8 hours	0.248	36° 8 hours	0.086
	39° 1 hour heat shock	0.878	39° 1 hour heat shock	0.988
**Patched 1**	36° 8 hours	**0.007**	36° 8 hours	0.155
	39° 1 hour heat shock	**0.02**	39° 1 hour heat shock	0.505
**Gli1**	36° 8 hours	**0.001**	36° 8 hours	0.099
	39° 1 hour heat shock	**p<0.01**	39° 1 hour heat shock	0.887
**Sox10**	36° 8 hours*	0.358	36° 8 hours	**p<0.01**
	39° 1 hour heat shock*	*0.048*	39° 1 hour heat shock	**p<0.01**

*Higher order model was moderately not significant (p=0.057). Posthoc significance should be interpreted with caution.

Figure S1: Rare, but extreme examples of observed craniofacial malformations We observed two cases of diprosopus where the embryo had duplicated facial structures (**A**). The posterior cranium and body axis were not duplicated. Both embryos were dissected from eggs incubated at 33°C for 12 days, several weeks apart from one another. We observed a third case of diprosopy where the midline eyes were not fully separated and the mouths joined at their base. We also collected two embryos with the complete loss or near-complete loss of midline facial structures, one of the most severe phenotypes observed in our study (**B, C**). In these embryos the forebrain (FB) is reduced to a small protuberance and the eyes have collapsed together at the midline. The midbrain (MB) and hindbrain (HB) appear normal. Both embryos were collected from gravid females maintained at 37°C, but whose eggs were incubated at 27°C.

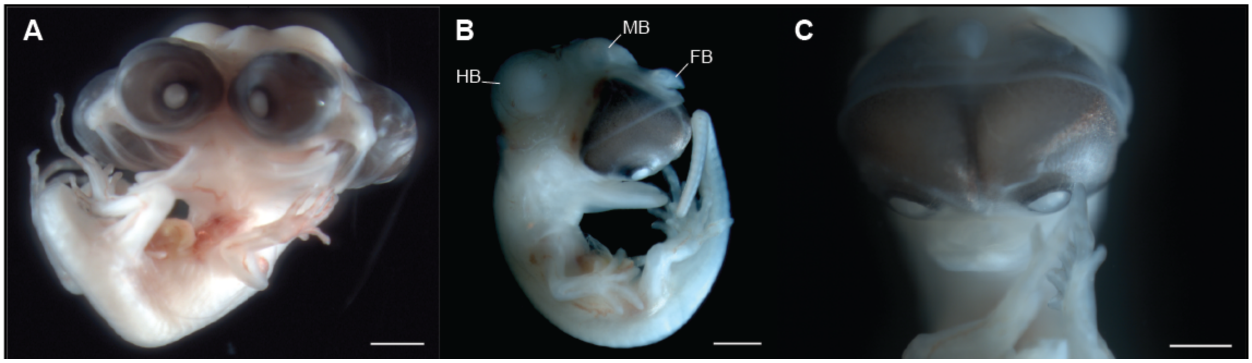

Figure S2: Positive correlation between maximum ground temperature and sun exposure
Increased sun exposure elevates the maximum ground temperature of potential nest sites (solid line, $p<0.001$). The minimal daytime temperature (hours 6-20) of each site does not change based on a site's sun exposure (dashed line, $p=0.40$). These results illustrate that nest sites with greater sun exposure will experience higher temperatures with greater fluctuations than sites with low sun exposure. Lux measurements represent the average measure of sun each site experienced over 10 hours of daylight observation. Red dots represent the three sites with the most sun exposure while blue represent the sites with the least sun exposure, paralleling what is depicted in Figure 2.

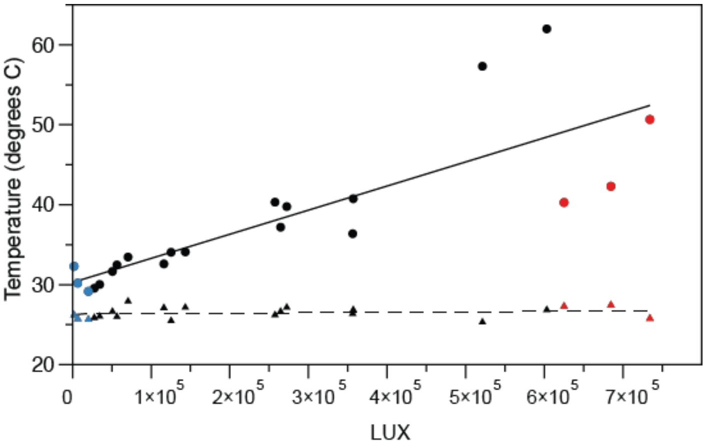

Figure S3: Examples of putative nest sites We collected temperature and sun exposure data (Lux) across a spectrum of putative natural nest sites, ranging from those with full sun exposure to those with significant cover. Brown anole females were observed in close proximity to all areas where thermal data was collected.

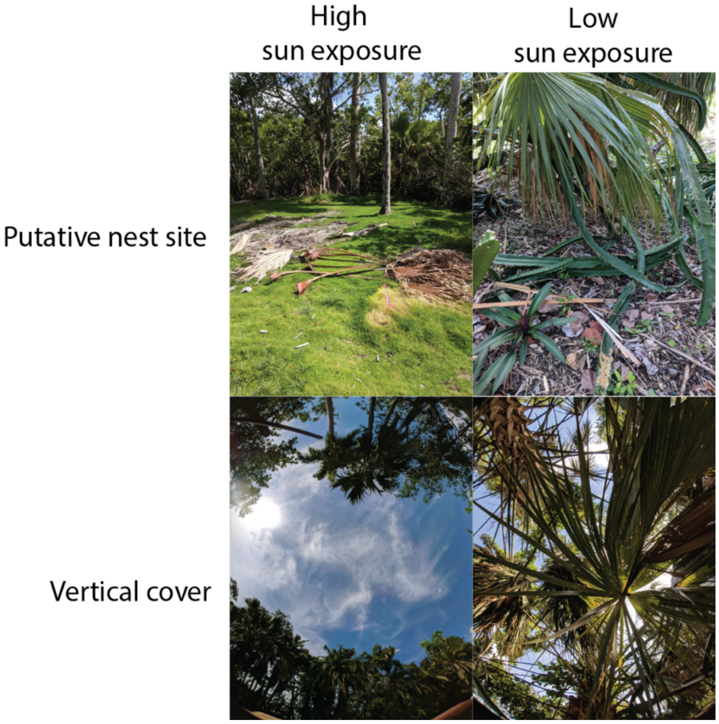

Figure S4: Stage-specific effects of Shh on *A. sagrei* facial development The effects of Shh on *A. sagrei* facial development are stage-specific. (A) Treatment of *A. sagrei* embryos with 100µM cyclopamine on the day of oviposition leads to a failure of midline facial process development (post-oviposition day three embryos, experimental embryo above control embryo). (B) In contrast, treatment of *A. sagrei* embryos on the second day of incubation, after the induction of the Shh in the oral ectoderm, leads to a hypoplastic face (above) compared to the control (below), by day 12. Mild facial clefting was observed in two out of six treated embryos exposed to 100µM cyclopamine on day 2.

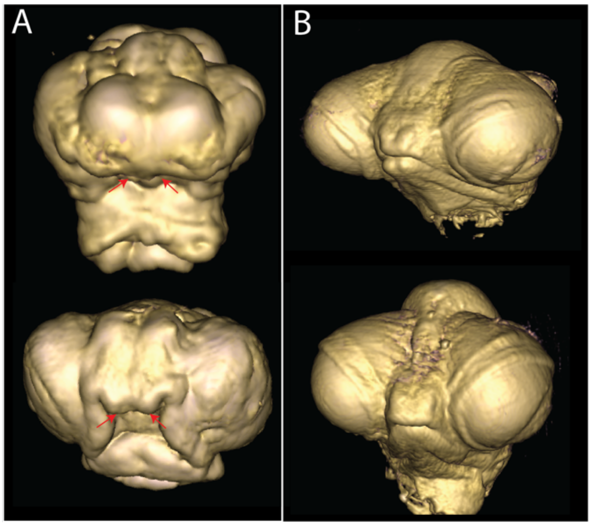

Figure S5: Stage specificity of induction of craniofacial malformations We tested the stage specificity of the developmental model with three incubation experiments that isolate the first 48 hours of post-oviposition development. Because the earliest stages of anole development, including fertilization, gastrulation, neurulation, and neural crest migration occur within the female we maintained gravid females at 36°C. In the wild today, body temperatures of gravid *A. sagrei* females reach at least 34°C (Sanger et al. 2018). The captive females behaved normally and laid eggs regularly. Maintaining females at this temperature moderately elevated the rate of malformation above baseline levels (N=88 incubated; N=53 surviving [dark bar]; 7.5% malformed [light bar]). Malformations were concentrated to the head and face. One individual exhibited the complete loss of the face and forebrain (Supp. Fig. 3). Based on this localized pattern we predict that these malformations were induced just prior to oviposition potentially due to cell death in the anterior neural tube. Embryos incubated at 36°C for the first 48 hours of development exhibited only moderate reduction in survival (N = 54 incubated, 84% survival), but of the surviving embryos 11% exhibited similar craniofacial malformation to those previously observed. Conversely, embryos incubated at 27°C for 48 hours and 36°C until day 12 did not exhibit craniofacial malformations, although their survival was much more dramatically impacted (N = 51 incubated; 51% survival).

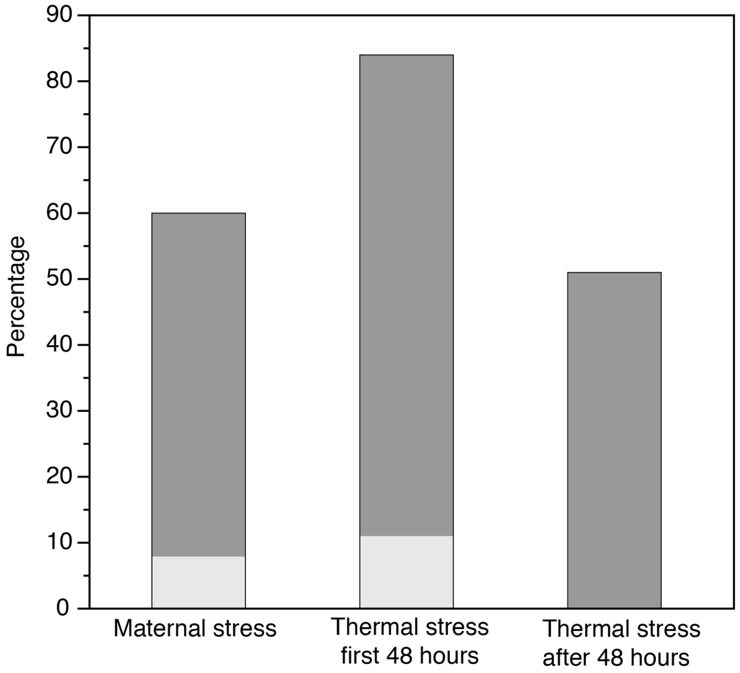

Supplement: obab033_Supplemental_File [file obab033_supplemental_file.pdf]
